# *Chenopodium botrys* Extract Affects Acute Kidney Injury Caused by Rhabdomyolysis in Rats Through TNF/NF‐κB Signaling Pathway

**DOI:** 10.1002/fsn3.4667

**Published:** 2024-12-10

**Authors:** Mohammad Amin Momeni‐Moghaddam, Abbasali Abbasnezhad, Amir Hossein Ebadi, Reza Mohebbati

**Affiliations:** ^1^ Department of Nutrition and Biochemistry, Faculty of Medicine, Social Determinants of Health Research Center Gonabad University of Medical Science Gonabad Iran; ^2^ Department of Physiology, Faculty of Medicine Gonabad University of Medical Sciences Gonabad Iran

**Keywords:** acute kidney injury, *Chenopodium botrys*, inflammation, rhabdomyolysis

## Abstract

Due to the anti‐inflammatory and antioxidant properties of the *Chenopodium botrys* and the pathological mechanisms of rhabdomyolysis in the kidney, this plant can be used to improve the symptoms of this disease. Then, in this study, we investigated the effects of this herb in improving kidney injury by rhabdomyolysis. Animals were divided into five groups: control, glycerol (received it for rhabdomyolysis induction), extract (received 12 mg/kg *C. botrys* extract), and treatment groups with dexamethasone (0.03 mg/kg) and extract (12 mg/kg). The extract was analyzed using HNMR. After a week, blood and urine samples were taken to measure protein, urea, and creatinine. Then, the animals were sacrificed, and the kidney tissue was removed to examine the antioxidant, TNF‐α, and histopathological evaluations. Also, NF‐κB gene expression was investigated. The serum creatinine, TNF‐α, and NF‐κB ratio significantly increased and antioxidant capacity decreased in the glycerol group compared with the control. Pathological evaluation also showed severe renal damage based on the related criteria. In the treatment groups with dexamethasone and especially extract, the considered parameters attenuated relatively compared with the glycerol group. Kidney damage and functional impairment associated with rhabdomyolysis, as well as the inflammatory response caused by increased NF‐κB and the proinflammatory cytokine TNF‐α, may be alleviated by *C. botrys* . Consequently, *C. botrys* could represent a potential therapeutic approach for patients with rhabdomyolysis‐induced acute kidney injury.

## Graphical abstract

The serum creatinine, TNF‐α, and NF‐κB expression increased and antioxidant capacity decreased in the kidney injury caused by rhabdomyolysis and then the pathological evaluations approved it. *Chenopodium botrys* extract administration decreased creatinine, TNF‐α, and NF‐κB expression and improved antioxidant system as well as pathological changes. TNF/NF‐κB signaling pathway is a possible mechanism for kidney injury improvement by extract.

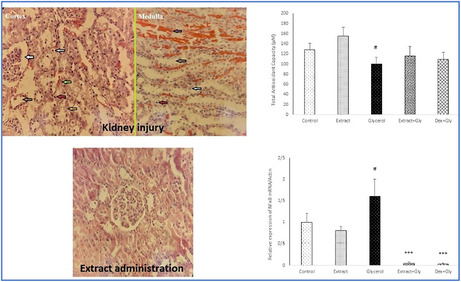

## 1 Introduction

Rhabdomyolysis means destruction or loss of muscle integrity. In this syndrome, the imbalance between energy production and consumption in myocytes causes ischemia and ultimately releases intracellular compounds to the extracellular environment (McMahon, Zeng, and Waikar 2013). Several studies have been conducted to classify the causes of rhabdomyolysis. Age, trauma, drug abuse, and infections are the most common causes of rhabdomyolysis in adults and children. Trauma is the cause of 85% of cases (Splendiani et al. 2001). Besides the degeneration of myofibrils, cocaine and heroin can also cause severe vasoconstriction of skeletal muscle arteries, muscle ischemia, and ultimately acute rhabdomyolysis by inhibiting the reabsorption of catecholamines in alpha‐adrenergic receptors (Gabow, Kaehny, and Kelleher 1982). Regardless of the underlying cause, damage to myocytes in rhabdomyolysis causes an imbalance between ATP production and consumption, mitochondrial dysfunction, increased production of free radicals, and ultimately cell death. Following the decrease in energy and dysfunction of the ATPase pump, the intracellular sodium level increases, which causes the activity of the sodium‐calcium exchanger, the increase of calcium ions in the cytoplasm of myocytes, and the activation of proteases and apoptotic pathways (Zimmerman and Shen 2013).

On the other hand, the damage of myocytes causes the activity of phospholipase A to change the viscosity of the sarcolemma membrane, increase membrane permeability, and release large amounts of potassium, phosphate, urate, lactate dehydrogenase, aspartate aminotransferases, alanine aminotransferases, aldolase, creatine kinase, and myoglobin into the bloodstream, which can cause capillary damage, edema, ischemia, cell necrosis, and metabolic acidosis (Boutaud and Roberts II 2011). The release of various mediators from damaged muscle cells such as nuclear DNA, mitochondrial DNA, and microRNA can also cause tubular damage in the kidneys by activating tumor necrosis factor‐alpha (TNF‐α), interleukin 6, 8, and 1‐beta (Panizo et al. 2015). Acute kidney injury (AKI) is the most common systemic complication of rhabdomyolysis, which is defined as an increase in serum creatinine more than 26.4 μmol/L during the first 48 h or an increase in serum creatinine more than 50% of the baseline value within 7 days after myocytes damage (Kellum and Lameire 2013). Although the exact pathogenesis of the development of AKI following rhabdomyolysis is not known, some mechanisms play a key role. In general, it can be said that oxidative damage (Parhizgar et al. 2016) and inflammation are the two main mechanisms in causing rhabdomyolysis. Therefore, antioxidant or anti‐inflammatory drugs can help to improve this disease.

Medicinal plants have a good position due to their easy access and having substances with antioxidant and anti‐inflammatory properties, among which *Chenopodium botrys* can be mentioned (Mohebbati et al. 2023). *C. botrys* has many effective substances such as phenol, flavonoid, and especially anthocyanin (de Pascual‐T et al. 1981). Due to the anti‐inflammatory and antioxidant properties of this plant and due to the pathological mechanisms of rhabdomyolysis in the kidney, this plant can be used to improve the symptoms of this disease. Then, in this study, we investigated the effects of the *C. botrys* extract in improving kidney damage by rhabdomyolysis.

## 2 Methods

### 2.1 Animal and Grouping

The present study was conducted on 25 adult male Wistar rats purchased from the laboratory animal center of Gonabad University of Medical Sciences weighing 200 ± 20 g. First, the animals were kept for 2 weeks to adapt to the environmental conditions with an average temperature of 23°C± 2°C and observe the light/dark cycle for 12 h each with free access to food and water. Then, the rats were randomly divided into five groups of five and placed separately in special cages:

Group 1 (control group): gavage of 1 mL of normal saline daily from days 1 to 7 of the study.

Group 2 (glycerol group): gavage of 1 mL of normal saline daily from days 1 to 7 of the study + injection of 10 mg/kg of 50% glycerol intramuscularly (Mahmood and Kathem 2023) on the third day of the study to cause rhabdomyolysis.

Group 3 (dexamethasone + glycerol group): gavage of 0.03 mg/kg of dexamethasone (Donald et al. 2003) daily from days 1 to 7 of the study + intramuscular injection of 10 mg/kg of 50% glycerol on the third day of the study to cause rhabdomyolysis.

Group 4 (extract + glycerol group): gavage of 12 mg/kg extract of *C. botrys* (determined by pilot study) daily from days 1 to 7 of the study + injection of 10 mg/kg 50% glycerol intramuscularly on the third day of the study to cause rhabdomyolysis.

Group 5 (extract group): gavage of 12 mg/kg hydroalcoholic extract of *C. botrys* daily from days 1 to 7 of the study.

### 2.2 Study Design

On days 0 and 6 of the study, animals were placed in a metabolic cage and their urine was collected for 24 h. Also, on days 0 and 7, urine and blood samples were taken to measure urea, urine creatinine, and blood serum. Blood samples were taken from the eyes on day 0 and from the heart on day 7 of animals under anesthesia with 100 ketamine and 10 mg/kg xylazine. To evaluate malondialdehyde (MDA) (Khazdair et al. 2016) and total antioxidant capacity (TAC), the right kidney of the animals was kept at −80°C after washing. The left kidney was kept in 10% formalin for histological tests. After paraffinization, they were cut into 3‐ to 4‐μm slices. The slices were stained with hematoxylin and eosin (H&E) for light microscopic analysis.

### 2.3 Parameters Analysis

To investigate the NF‐κB gene expression, RNA extraction from kidney tissue cells was performed. Determining the purity of extracted RNA as well as determining its concentration is done by measuring the absorption ratio of A260/A280 using a nanodrop device. Then, the reverse transcription reaction is performed and cDNA is made from the extracted RNA. The concentration of cDNA was measured by the photometric method and the amount of NF‐κB gene expression was measured using specific primers and the real‐time PCR technique.

The ELISA method is used to measure TNF‐α. The principles of ELISA are the same as the basic principles of immunology, that is, the specific binding of antigens and antibodies. In the sandwich ELISA method to identify the specific antigen (TNF‐α), a specific antibody is attached to the bottom of the container, and to prevent the nonspecific binding of the rest of the container, it is covered with bovine serum albumin, and by adding the liquid containing the antigen to be measured, the antigen is added to the antibody. The bottom of the container is connected, and after washing, the primary antibody is added that recognizes another epitope of the antigen and takes the antigen together with the bottom of the container (sandwich), after washing, the secondary antibody is added that contains a specific enzyme (horseradish peroxidase [HRP]). HRP is attached to it and this secondary antibody is attached to the primary antibody, after washing the added enzyme substrate, which is converted by the enzyme into a product that creates a different color from the substrate. Quantitative measurement is done based on this color change.

To identify some ingredients of the extract, the proton nuclear magnetic resonance (HNMR) made by Bruker instrument (Germany) was used. The extract dissolved in deuterated DMSO (to prevent the creation of disturbing proton peaks in the NMR spectrum) was placed in a special NMR tube and then placed in the magnetic field of the NMR instrument.

### 2.4 Statistical Analysis

The information was recorded in the set checklist, and after the end of the study, the information was entered into the Instat, to compare the variables between different groups, after confirming the normality by the Kolmogorov–Smirnov test, the one‐way analysis of variance statistical test following Tukey's post hoc used. Significance was considered < 0.05.

## 3 Results

**GRAPH 1:**
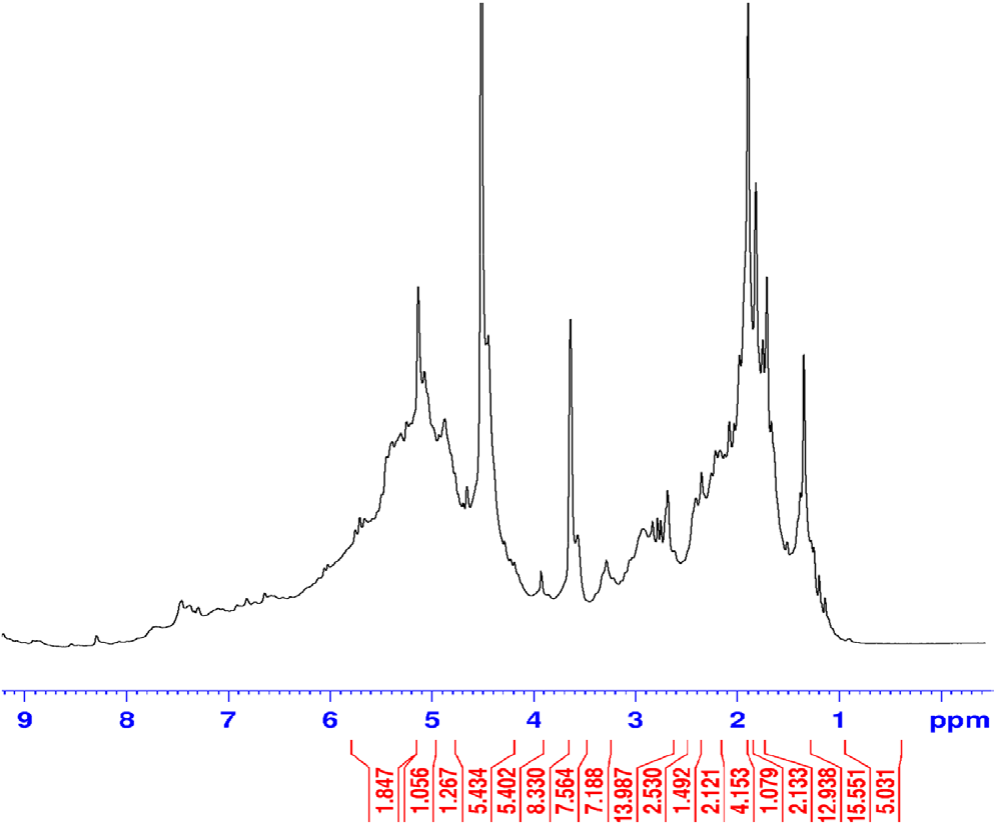
H NMR spectrum of the extract. The bottom line depicts the values of the integral areas.

*C. botrys* was analyzed using HNMR as following: In the region below five, there are vinyl C=C groups (i.e., CH groups attached to a double bond). There are also alkane CH groups (i.e., CH groups with SP3 hybridization), indicating a large presence of aliphatic compounds. In the region around four, there are peaks corresponding to O‐methyl and O‐ethyl groups, and their concentration is high. In the region around three, hydrogens are observed attached to carbons that are connected to nitrogen (i.e., CH groups attached to an N). In the aliphatic region, a large quantity of compounds are present while in the aromatic region compounds different is less. Ester compounds were also found in the extract. The CH_2_ groups present are related to aliphatic compounds. Additionally, CH groups attached to NO_2_ were found in the region around four, accompanied by O‐ethyl and O‐methyl groups (Graph 1).

**FIGURE 1:**
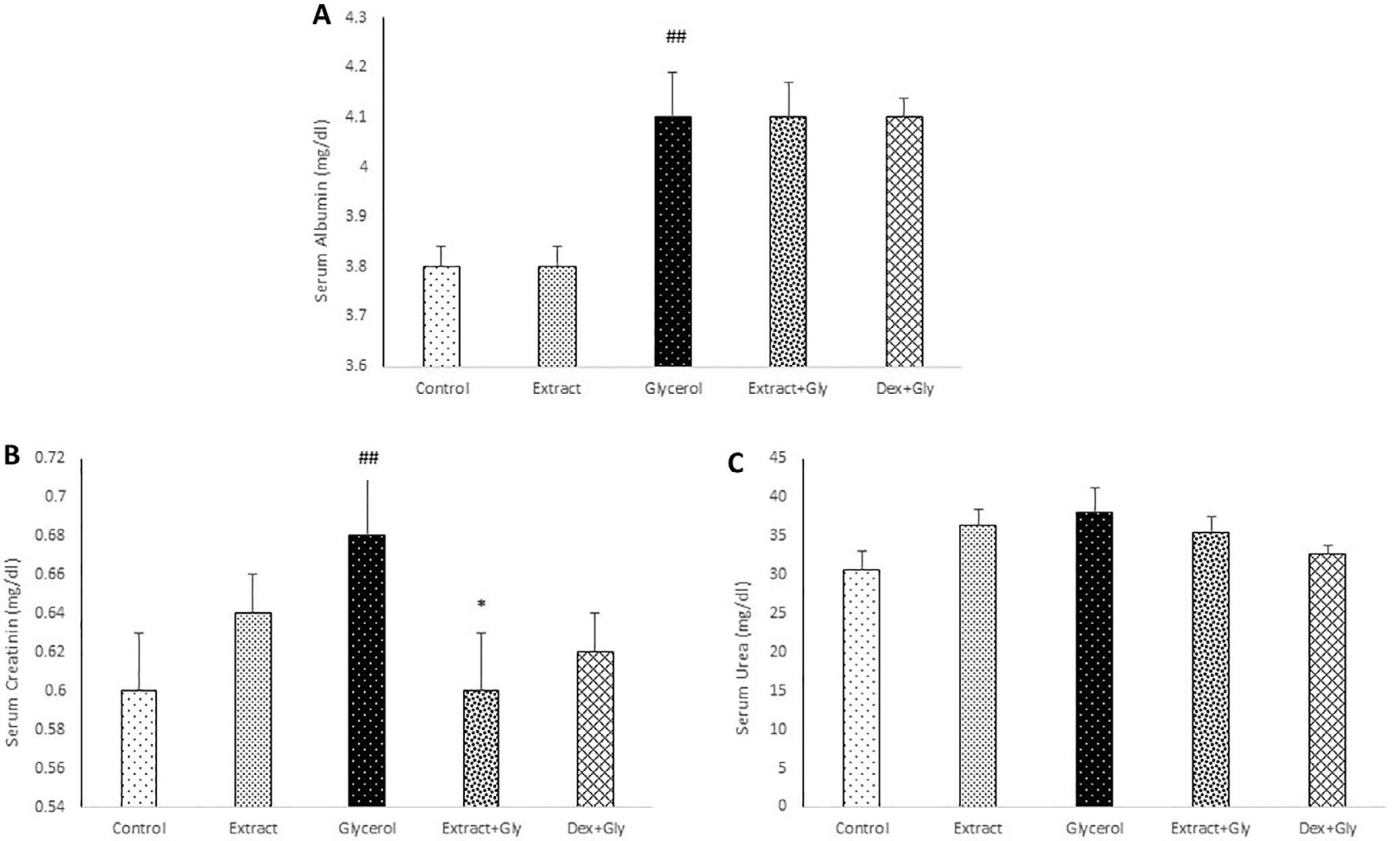
Comparison of the serum albumin (A), creatinine (B), and urea (C) in experimental groups. Data are presented as mean ± SEM (*n* = 5 in each group). ^##^ *p* < 0.01 compared with the control group. *p < 0.05 compared with the glycerol group.

Serum albumin, urea, and creatinine significantly increased in the glycerol group compared with the control (*p* < 0.01), while the creatinine level significantly decreased in the treatment group with extract compared with the glycerol group (*p* < 0.05) (Figure 1).

**FIGURE 2:**
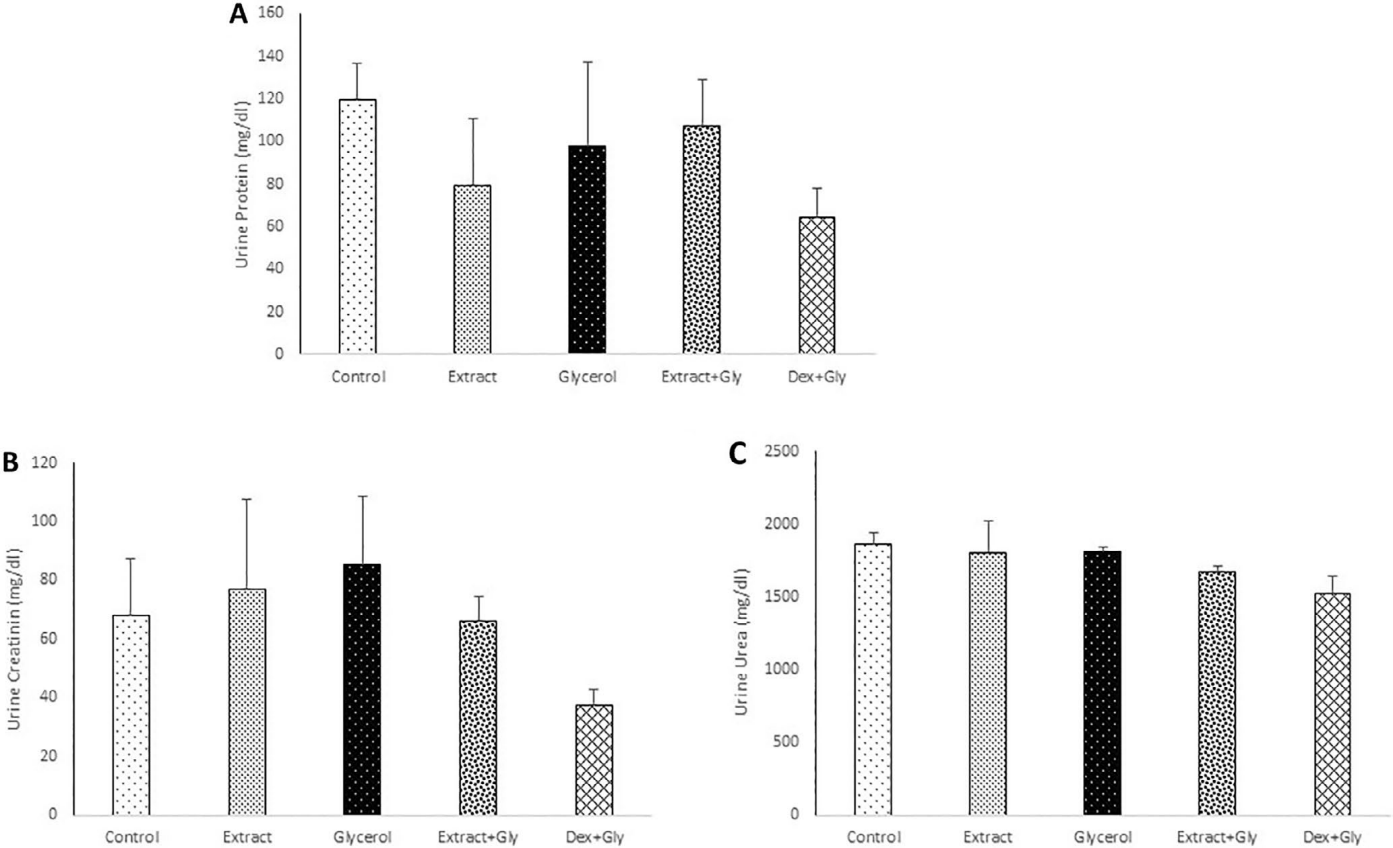
Comparison of the urine protein (A), creatinine (B), and urea (C) in experimental groups. Data are presented as mean ± SEM (*n* = 5 in each group).

Changes in the urine protein, urea, and creatinine were not significant in any groups (Figure 2).

**FIGURE 3:**
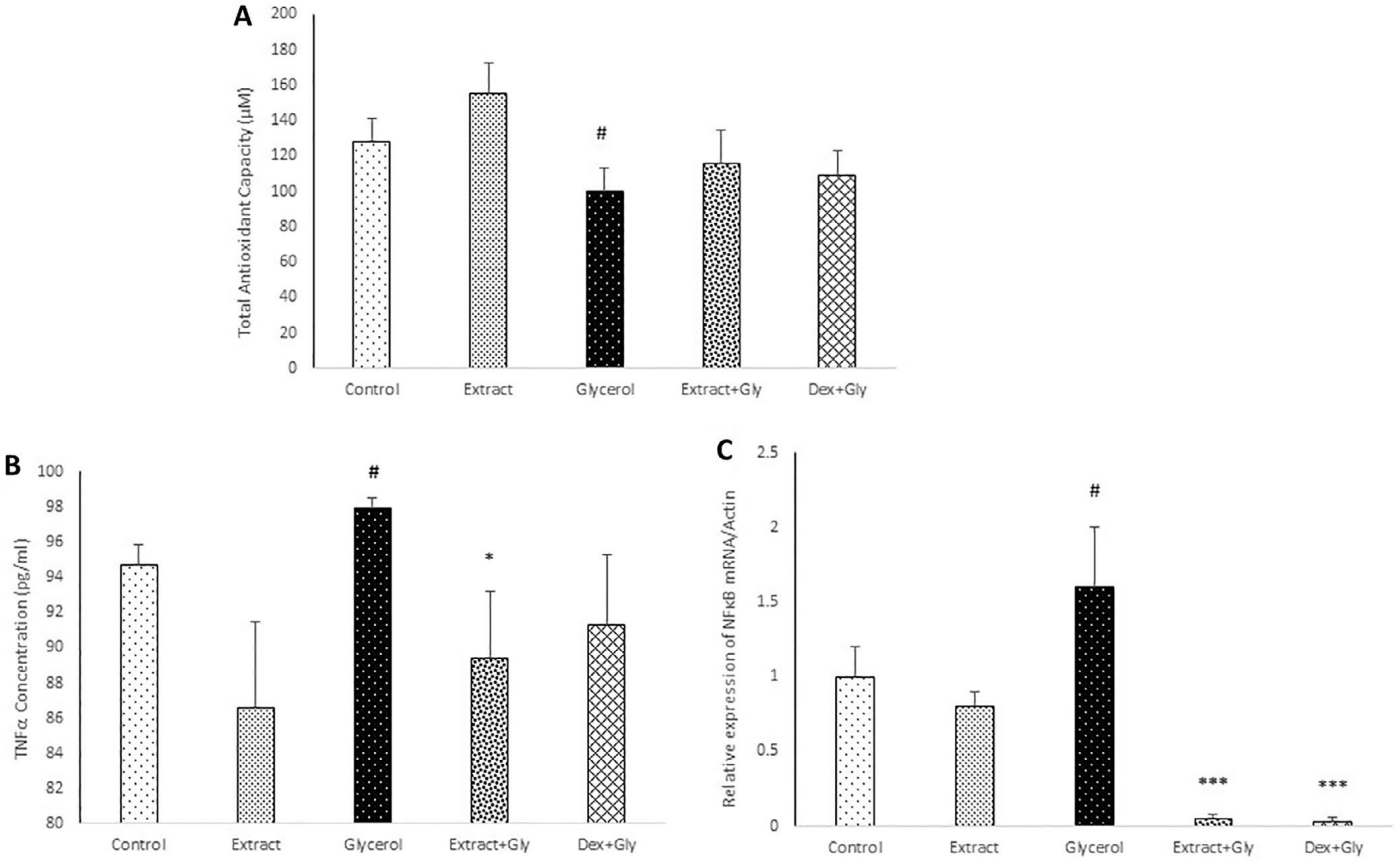
Comparison of the total antioxidant capacity (A), NF‐κB ratio (B), and TNF‐α level (C) in experimental groups. Data are presented as mean ± SEM (*n* = 5 in each group). ^#^ *p* < 0.05 compared with the control group. *p < 0.05 and ***p < 0.001 compared with the glycerol group.

In the glycerol group, the TAC significantly decreased compared with the control (*p* < 0.05), while in treatment groups with extract and dexamethasone, this parameter decreased nonsignificantly compared with the glycerol group (Figure 3A).

The TNF‐α serum level significantly increased in the glycerol group compared with the control (*p* < 0.05), while in the treatment group with extract, this parameter significantly reduced compared with the glycerol group (*p* < 0.05) (Figure 3B).

The NF‐κB ratio in the glycerol group significantly boosted in comparison with the control (*p* < 0.05), while in treatment groups with extract and dexamethasone, this parameter significantly decreased compared with the glycerol group (*p* < 0.001) (Figure 3C).

**FIGURE 4:**
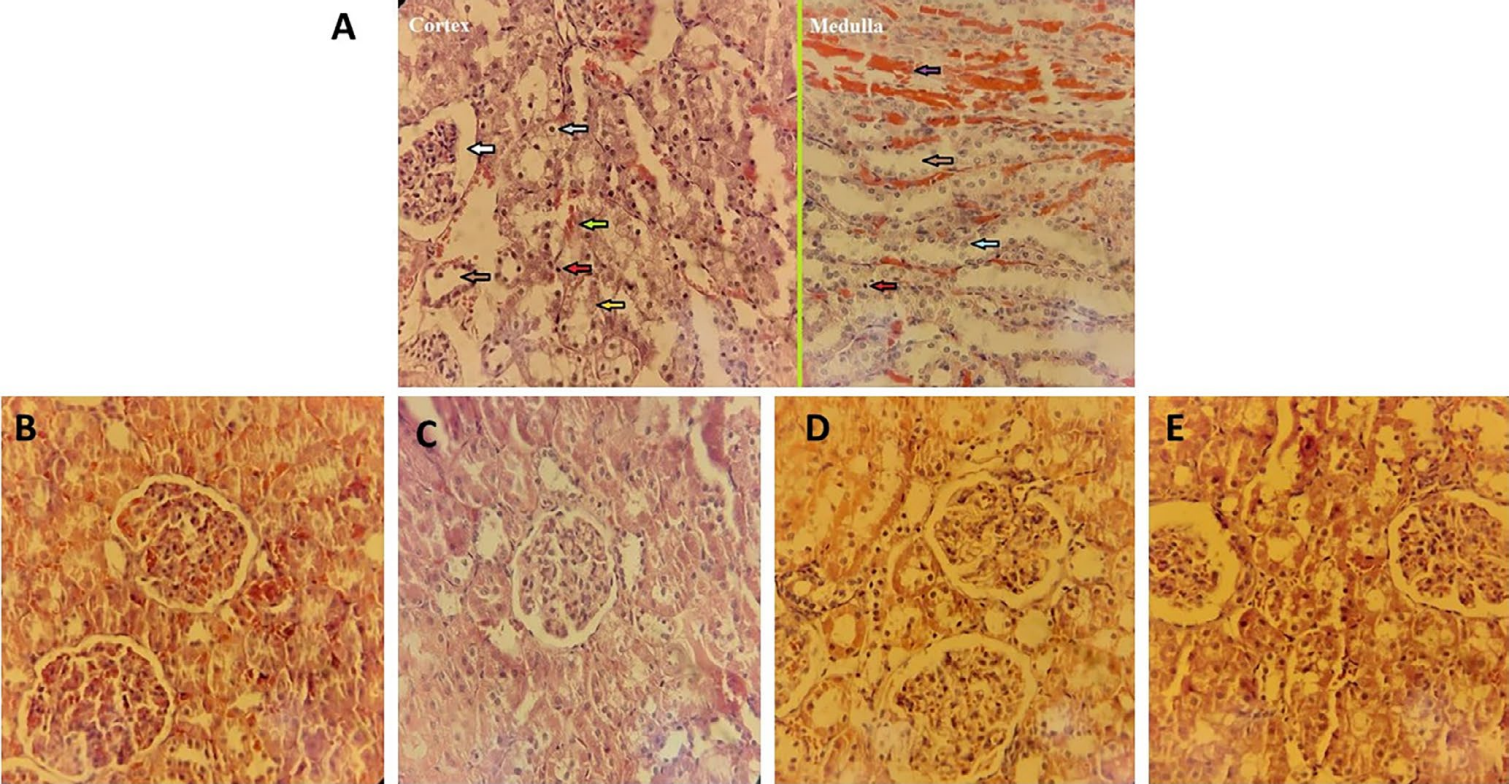
Sections of renal tissues stained with hematoxylin–eosin. (A) Glycerol group (cortex and medulla), (B) control group, (C) extract receiving group, and (D and E) treatment groups with extract and dexamethasone, respectively. The white arrow indicates glomerular/tubule granular degeneration, the brown arrows indicate tubule dilation, the gray arrow indicates cell vacuolization, the green arrow indicates hemorrhage/congestion, the red arrows indicate tubule pyknotic nucleus, the yellow arrow indicates tubule karyolitic nucleus, the blue arrow indicate tubule karyorrhexis nucleus, and the purple arrow indicates myoglobin cast. × 40 magnification.

Pathological evaluation indicated that in the glycerol group, the injury was induced through glomerular/tubule granular degeneration, tubule dilation, cell vacuolization, hemorrhage/congestion, tubule apoptotic nucleus, and myoglobin cast. In the treatment group with extract and dexamethasone, these injury criteria attenuated (Figure 4).

## 4 Discussion

The current study aimed to investigate the effects of *C. botrys* plant extract on NF‐κB expression, TNF‐α serum levels, and TAC in a model of AKI induced by rhabdomyolysis. Glycerol was used to induce rhabdomyolysis, with dexamethasone serving as the positive control. The findings of the present research indicated that treatment with *C. botrys* extract significantly reduced NF‐κB expression and serum TNF‐α levels in rats compared with those treated with glycerol. While the TAC in glycerol‐treated rats decreased significantly compared with the control group, the *C. botrys* ‐treated group showed an increase in TAC, though this increase was not statistically significant compared with the glycerol group.

Rhabdomyolysis is a pathological condition characterized by the breakdown of skeletal muscles and the release of intracellular contents into the bloodstream, often resulting in AKI (Baatarjav et al. 2022). Research suggests that inflammation plays a critical role in the pathophysiology of rhabdomyolysis‐induced AKI (Komada et al. 2015), with the NF‐κB pathway being a key contributor (Guerrero‐Hue et al. 2019). Oxidative stress and inflammatory processes triggered by glycerol can activate the NF‐κB pathway (Reis et al. 2019). Once activated, this signaling pathway promotes the production of proinflammatory cytokines such as TNF‐α during AKI (Amirshahrokhi 2021).

Muscle cell damage caused by rhabdomyolysis releases immunostimulatory molecules, which reach the kidneys and activate the NF‐κB signaling pathway. Increased NF‐κB expression contributes significantly to the progression of glycerol‐induced AKI by driving the synthesis of inflammatory mediators, such as macrophages and T lymphocytes, which are strongly correlated with both kidney dysfunction and morphological changes. The activation of these inflammatory cells leads to the production of proinflammatory cytokines, including TNF‐α, thereby sustaining a proinflammatory environment (Liu et al. 2017; Shimizu et al. 2023).

Immunohistochemical analyses in this study demonstrated that glycerol‐induced rhabdomyolysis led to AKI, accompanied by increased inflammation. This inflammatory response is largely attributed to the activation of NF‐κB, a key transcription factor in inflammatory pathways. Following NF‐κB activation, the expression of the TNF‐α gene was upregulated. However, in groups that received *C. botrys* extract or dexamethasone alongside glycerol, NF‐κB expression was significantly reduced compared with the glycerol‐only group, indicating the strong anti‐inflammatory effects of these compounds. It is well‐established that dexamethasone, as a corticosteroid, exerts potent anti‐inflammatory effects by inhibiting NF‐κB (Aghai et al. 2006; Davies, Adlimoghaddam, and Albensi 2021). Similarly, *C. botrys* extract demonstrated a comparable reduction in NF‐κB expression, suggesting the presence of active anti‐inflammatory compounds within the plant that modulate inflammatory responses by inhibiting the NF‐κB pathway. This reduction in NF‐κB also leads to decreased production of inflammatory cytokines, such as TNF‐α, reduces oxidative damage, and improves kidney function. Supporting this (Bojilov et al. 2023), found that *C. botrys* is rich in flavonoids, especially 6‐methoxy flavones, compounds known for their anti‐inflammatory properties. In another study by (Zhong et al. 2022) demonstrated that flavonoids inhibit the NF‐κB signaling pathway, thus reducing inflammation. Consequently, the rats treated with *C. botrys* extract, like those treated with dexamethasone, were able to effectively reduce both inflammation and kidney damage through NF‐κB inhibition.

The current study demonstrated that glycerol‐induced AKI significantly increased serum TNF‐α levels, which were markedly reduced following treatment with *C. botrys* . TNF‐α, a proinflammatory cytokine, is typically expressed in response to injury and inflammation, subsequently promoting the release of additional inflammatory cytokines (Wu et al. 2017). Rhabdomyolysis‐induced muscle damage leads to the release of myoglobin into the bloodstream, which causes kidney damage, generates free radicals, and elevates proinflammatory cytokines such as TNF‐α. This cascade exacerbates the inflammatory response and further damages kidney tissue (Panizo et al. 2015). In the glycerol‐treated rats, serum TNF‐α levels significantly increased compared with the control group. However, rats treated with *C. botrys* extract in addition to glycerol exhibited a marked reduction in TNF‐α levels compared with the glycerol‐only group, confirming the plant's anti‐inflammatory efficacy. Active compounds within *C. botrys* , such as flavonoids and polyphenols, may help mitigate inflammation by inhibiting pathways like NF‐κB and reducing proinflammatory cytokines such as TNF‐α. In contrast, although TNF‐α levels decreased in the glycerol and dexamethasone group, the reduction was not statistically significant. Studies have shown that TNF‐α interacts with the NF‐κB signaling pathway, activating it and thereby promoting the expression of other inflammatory cytokines, which amplifies the inflammatory response in AKI (Yu et al. 2015). TNF‐α‐mediated NF‐κB activation triggers phosphorylation and degradation of IκB in the cytoplasm, enabling the NF‐κB p65 subunit to translocate to the nucleus, where it regulates target gene expression (Lee et al. 2017). Based on these observations, the reduction in NF‐κB expression in the *C. botrys* group may be primarily due to TNF‐α inhibition, while in the dexamethasone group, a different mechanism may be responsible. One potential alternative pathway is IL‐1β, which can also activate NF‐κB independently of TNF‐α (Guo et al. 2024). It is also possible that the dose of dexamethasone used in this study was insufficient to significantly reduce TNF‐α levels, or that dexamethasone exerts its effects via other inflammatory pathways and proinflammatory cytokines. Hence, dexamethasone demonstrated anti‐inflammatory effects through NF‐κB suppression, though the reduction in TNF‐α was not statistically significant in this study, possibly due to the dosage or its varied mechanisms of action.

Oxidative stress plays a crucial role in the pathogenesis of myoglobinuric AKI induced by rhabdomyolysis (Gyurászová et al. 2020). The rise in free radicals depletes the body's antioxidant reserves, reducing TAC. A decrease in TAC reflects an imbalance between free radical production and the body's ability to neutralize them. Oxidative stress triggers NF‐κB activation, a pathway initiated by ROS (reactive oxygen species) or other stimuli that phosphorylate IκBα. Phosphorylated IκBα undergoes degradation, releasing NF‐κB to enter the nucleus and regulate genes involved in inflammation and oxidative responses (Hong et al. 2024; Kim et al. 2010; Lingappan 2018). Elevated NF‐κB activity upregulates gene expression, including that of NADPH oxidases (NOXs) (Liao et al. 2023; Wang et al. 2022). Wang et al. (2022) demonstrated in vivo that Ulin statin, an anti‐inflammatory and free radical scavenger, attenuates myoglobin‐induced cytotoxicity by suppressing intracellular ROS via the TLR4/NF‐κB signaling pathway. NADPH oxidases (NOXs) are among the factors that can be upregulated by NF‐κB (Gauss et al. 2007; Sedeek et al. 2013; Wu et al. 2021). NOXs, a family of membrane‐bound enzymes, primarily generate ROS (Bevilacqua et al. 2012; Cipriano et al. 2023; Magnani and Mattevi 2019). Studies indicate that polyphenols and flavonoids inhibit ROS production through the NF‐κB/NADPH oxidase pathway (Andrés et al. 2023; Andriantsitohaina et al. 2012; Maraldi 2013; Slika et al. 2022). NF‐κB activation and subsequent ROS elevation increase the demand for antioxidants, potentially depleting TAC and compromising cellular components such as lipids, proteins, and DNA (Feng et al. 2016). This process exacerbates inflammation and chronic diseases associated with oxidative stress, including rhabdomyolysis. Glycerol‐induced rhabdomyolysis leads to muscle cell damage, releasing myoglobin into the bloodstream and causing kidney damage. This process also results in the excessive production of free radicals, increasing oxidative stress. Fernández‐Fúnez et al. (2003) observed decreased total serum antioxidant status in a glycerol‐induced myoglobinuric‐rhabdomyolysis model of AKI. In this study, TAC levels significantly decreased in the glycerol‐treated group compared with the control group. A similar outcome was observed in a study by (Ahmed et al. 2022), where glycerol treatment reduced TAC in rats. Also, (Fernández‐Fúnez et al. 2003) observed decreased total serum antioxidant status in the glycerol‐induced myoglobinuric‐rhabdomyolysis model of AKI. However, mice treated with *C. botrys* extract showed increased TAC levels compared with the glycerol group. Flavonoids and other polyphenols possess an optimal chemical structure for scavenging free radicals and enhancing TAC due to their antioxidant properties (Csepregi et al. 2016). Given that NF‐κB activation correlates directly with ROS and oxidative stress, this pathway's inhibition potentially increases TAC. Compounds found in *C. botrys* , such as flavonoids and polyphenols, likely decrease ROS and enhance TAC via the NF‐κB/NADPH oxidase pathway.

Additionally, this study showed that serum creatinine levels in the glycerol‐treated group increased significantly compared with the control group. In contrast, rats treated with *C. botrys* extract and glycerol experienced a significant reduction in serum creatinine levels compared with the glycerol‐only group. Rhabdomyolysis‐induced AKI is known to elevate serum creatinine levels, as demonstrated by (Guerrero‐Hue et al. 2019), where rhabdomyolysis in rats led to an increase in serum creatinine. Creatinine is a widely used marker for assessing kidney function, and elevated levels in the glycerol group indicate a decline in glomerular filtration function due to kidney damage. In rhabdomyolysis, the mass release of creatinine into the bloodstream, coupled with kidney injury, results in creatinine accumulation as the kidneys are unable to effectively filter it. The elevated creatinine levels signify impaired glomerular filtration, which directly stems from kidney cell damage and reduced kidney function (Walid 2008). The significant reduction in creatinine levels in rats treated with *C. botrys* extract suggests that the plant may have protective effects on the kidneys, improving their function in the face of rhabdomyolysis‐induced damage. In the end, the pathological findings also demonstrated the improver effects of the extract on renal damage.

## 5 Conclusion

In summary, the results of the present study demonstrated that kidney tissue damage and functional impairment associated with rhabdomyolysis, as well as the inflammatory response caused by increased NF‐κB and the proinflammatory cytokine TNF‐α, may be alleviated by *C. botrys* . Consequently, *C. botrys* could represent a potential therapeutic approach for patients with rhabdomyolysis‐induced AKI.

## Author Contributions

R.M. conceptualization, methodology; A.A. and A.H.E. visualization, investigation; M.A.M.‐M. data curation, writing – original draft preparation; M.A.M.‐M. and R.M. reviewing and editing.

## Ethics Statement

All animal procedures based on the ARRIVE guidelines were under ethical license ID: IR.GMU.REC.1401.160. It was approved by the Gonabad University of Medical Sciences, Iran.

## Conflicts of Interest

The authors declare no conflicts of interest.

## Data Availability

The data that support the findings of this study are available from the corresponding author upon reasonable request.
